# Anti-Obesity and Anti-Inflammatory Synergistic Effects of Green Tea Catechins and Citrus β-Cryptoxanthin Ingestion in Obese Mice

**DOI:** 10.3390/ijms24087054

**Published:** 2023-04-11

**Authors:** Kazuhiko Nakadate, Kiyoharu Kawakami, Noriko Yamazaki

**Affiliations:** 1 Department of Basic Science, Educational and Research Center for Pharmacy, Meiji Pharmaceutical University, 2-522-1, Noshio, Kiyose, Tokyo 204-8588, Japan; d236953@std.my-pharm.ac.jp; 2 Department of Community Health Care and Sciences, Meiji Pharmaceutical University, 2-522-1, Noshio, Kiyose, Tokyo 204-8588, Japan; nyamazak@my-pharm.ac.jp

**Keywords:** green tea, catechin, β-cryptoxanthin, polyphenol, flavonoid, β-carotene, obesity, white adipose tissue, M1 macrophage, M2 macrophage

## Abstract

Chronic obesity causes various diseases, leading to an urgent need for its treatment and prevention. Using monosodium-glutamate-induced obesity mice, the present study investigated the synergistic obesity-reducing effects of tea catechins and the antioxidant β-cryptoxanthin present in mandarin oranges. The results show that the obese mice that ingested both tea catechin and β-cryptoxanthin for 4 weeks had a significantly decreased body weight, with no difference in body weight compared with control mice. Moreover, the blood biochemical test results were normal, and the body fat percentage was significantly decreased according to the histopathological analysis. Additionally, the abundance of M1 macrophages, which release pro-inflammatories, was significantly reduced in adipose tissue. Indeed, a significant decrease was detected in M1-macrophage-secreted tumor necrosis factor-alpha levels. Meanwhile, M2 macrophage levels were recovered, and adiponectin, which is released from adipocytes and involved in suppressing metabolic syndrome, was increased. Collectively, these results suggest that the combination of tea catechins and antioxidant foods can alleviate chronic obesity, indicating that a combination of various ingredients in foods might contribute to reducing chronic obesity.

## 1. Introduction

Obesity has recently become a major cause of metabolic syndrome worldwide [1,2]. Moreover, the incidence of obesity has more than doubled in children and quadrupled in adolescents in the past 30 years [3,4]. Children and adolescents with obesity are likely to experience obesity as adults; therefore, they are at an increased risk of adult health problems, such as heart disease, cardiovascular disease, type 2 diabetes, stroke, and osteoarthritis [5,6,7]. Additionally, chronic obesity is associated with an increased risk of cancer, including cancer of the breast, colon, endometrium, esophagus, kidney, pancreas, gall bladder, thyroid, ovary, cervix, and prostate, as well as multiple myeloma and Hodgkin’s lymphoma [8].

In obesity research, several studies have been conducted on animal models, as well as in humans. The onset of chronic obesity in humans can be caused by the excessive intake of high-calorie diets. Therefore, many associated studies co-induce obesity and type 2 diabetes in experimental animals by feeding them a high-calorie diet [9,10,11]. However, the data from these studies vary due to differences in diet. Therefore, monosodium glutamate (MSG) is often used to induce obesity [12,13], including in our previous studies [14,15,16]. In our previous studies on mice with MSG-induced obesity, we found that lipid droplets accumulated in the hepatocytes in the obese mice [15,16]. Other studies have also reported that lipid droplets accumulate in hepatocytes in chronic obesity and non-alcoholic fatty liver disease [17]. Moreover, our recent scanning electron microscopic analysis of the livers of mice with MSG-induced obesity showed sinusoidal dilatation and swelling of the sinusoidal fenestrations [15]. These studies suggest that obesity significantly affects not only blood cholesterol levels but also tissue structures, including that of the liver. Studies on these animal models have contributed to elucidation of the pathology of chronic obesity at the tissue and cellular levels. Increased adipose tissue is prominent in chronic obesity. Generally, adipose tissue is classified as an endocrine organ that produces and secretes various adipocytokines (physiologically active substances), such as adiponectin, leptin, and tumor necrosis factor-alpha (TNF-α), in addition to accumulating energy as neutral fat [18,19]. Macrophages distributed in adipose tissue and involved in inflammation can be divided into two subtypes: M1 macrophages and M2 macrophages [20,21]. The two primary types of macrophages (i.e., M1 and M2) exhibit different properties in adipose tissue [22]. M1 macrophages, which increase with obesity, secrete many pro-inflammatory cytokines and promote inflammatory changes in adipose tissue. In non-obese adipose tissue, M2 macrophages suppress inflammatory changes by producing anti-inflammatory cytokines.

Many studies on the prevention and alleviation of obesity have been reported, some of which have focused on green tea ingredients. Tea catechin, a type of polyphenol contained in green tea, exerts an antioxidant effect [23,24]. Active oxygen causes aging of the human body, age spots, wrinkles, and lifestyle-related diseases, such as arteriosclerosis. Hence, it is expected to exert a preventive effect against lifestyle-related diseases. Other effects of tea catechins on the human body include the lowering of blood cholesterol, suppression of blood pressure elevation, and suppression of blood glucose elevation [25,26,27]. Furthermore, ingesting tea catechins has been reported to moderate fat absorption [28,29]. In a human study, people who were obese to moderately overweight began to lose weight gradually when they drank approximately 200 mg of green tea containing catechins twice per day with breakfast and dinner for 12 weeks [30,31]. A difference in weight was observed between these individuals and those who drank beverages without catechins. Furthermore, the ingestion of beverages containing catechins has been reported to result in a decrease in not only body weight but also abdominal fat [31,32]. However, since it is difficult to consume enough catechins from tea alone each day, a need exists to identify other nutrients that exert a synergistic effect with catechins and improve obesity.

β-Cryptoxanthin reportedly exerts anticarcinogenic effects, exhibits preventive effects against osteoporosis, and lowers uric acid levels, primarily through its antioxidant effect [33,34,35]. It is a carotenoid present in the peel and flesh of citrus fruits, such as mandarin orange, persimmon, red pepper, papaya, and loquat. Along with α-carotene, β-carotene, lycopene, zeaxanthin, and lutein, it is retained in the human blood after ingestion [36,37]. In our previous animal study, following β-cryptoxanthin ingestion, its serum concentration increased in a dose-dependent manner [38]. β-Cryptoxanthin has also been shown to exhibit the ability to scavenge free radicals, reduce active oxygen, and suppress the production of nitric oxide in the body [39]. In addition, a cohort survey was conducted in people with a high intake of β-cryptoxanthin in the production area of mandarin oranges. According to the results from this study, β-cryptoxanthin is expected to exhibit various preventive effects that reduce the risk of lifestyle-related diseases [36,40].

Although studies have been conducted on the bioregulatory functions of individual food components contained in meals, the expected combination effect of a wide variety of food factors ingested simultaneously on bioregulatory functions remains unclear. Therefore, in the current study, we investigated the inhibitory effects of the simultaneous intake of tea components and β-cryptoxanthin on chronic obesity.

## 2. Results

### 2.1. Changes in Body Weight

**Figure 1:**
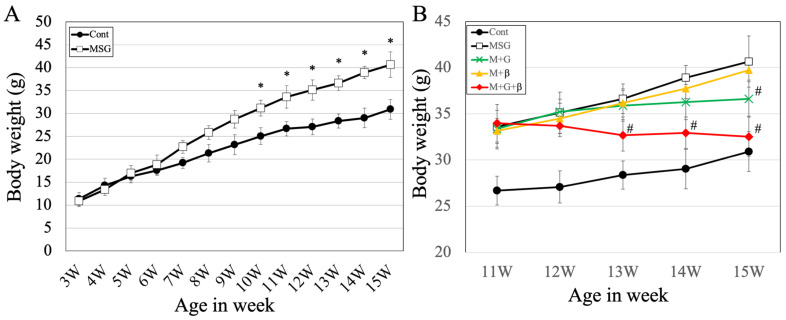
(**A**) Growth curve of the control and monosodium glutamate (MSG)-treated mice from postnatal day 3 to 15 weeks of age. Data are presented as mean ± standard deviation (*n* = 8 mice/group). * *p* < 0.05 compared to controls. (**B**) Body weight change over different drinking periods. Data are presented as mean ± standard deviation. *# p* < 0.05 compared to MSG-treated mice. Cont, MSG, M+G, M+β, and M+G+β indicate the control group, MSG-treated group, MSG-treated + green-tea-intake group, MSG-treated + β-cryptoxanthin-intake group, and MSG-treated + green tea + β-cryptoxanthin-intake group, respectively. The data are shown as the mean ± standard deviation values.

A mouse model of chronic obesity was generated by postnatal MSG administration, which is a method widely used to develop chronic obesity rodent models. We additionally reported histological changes in liver and gastrointestinal epithelial cells using an MSG-induced mouse model of chronic obesity [14,15]. The MSG-administered group showed an increase in average body weight from approximately 7 weeks of age compared to the control group and exhibited significant weight gain after 10 weeks of age (Figure 1). Water alone or water containing green tea catechin, β-cryptoxanthin, or green tea catechin and β-cryptoxanthin were administered for 4 weeks from 11 weeks of age (Figure 1B). Weight gain continued in the water-only group and the group administered β-cryptoxanthin-containing water. Meanwhile, those that consumed green-tea-catechin-containing water exhibited a slowed weight gain, and after 4 weeks, their weight decreased significantly compared with that of the chronically obese mice that were normally reared. Mice that drank green tea catechin and β-cryptoxanthin-containing water for 4 weeks showed a significant difference in body weight gain after two weeks of treatment compared to the mice with chronic obesity raised normally. Furthermore, after 4 weeks of drinking, no difference was observed between the average body weights of these mice and normally developing mice.

### 2.2. Food and Water Intake

A group of 11-week-old chronically obese mice showed weight loss after drinking water. We examined whether the cause of this weight loss was due to the amount of food and water intake. All MSG-administered groups tended to slightly increase food intake compared to the control group; however, the difference was insignificant (Table 1). Furthermore, no significant difference was observed in food intake among any groups, including the control group, nor in water intake (Table 2).

### 2.3. Blood Biochemistry Analysis

**Figure 2:**
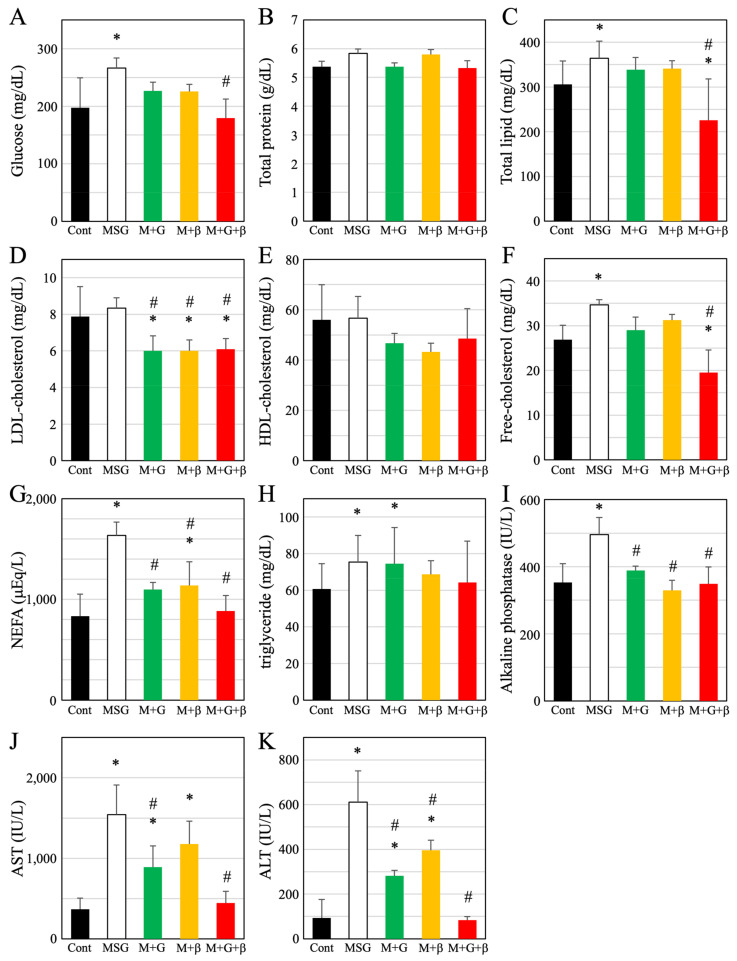
Blood chemistry values. (**A**) Blood glucose, (**B**) total protein, (**C**) total lipid, (**D**) low-density lipoprotein (LDL) cholesterol, (**E**) high-density lipoprotein (HDL) cholesterol, (**F**) free cholesterol, (**G**) free fatty acid (non-esterified fatty acid; NEFA), (**H**) triglyceride (TG; neutral fat), (**I**) alkaline phosphatase (ALP), (**J**) aspartate aminotransferase (AST), and (**K**) alanine aminotransferase (ALT). Cont, MSG, M+G, M+β, and M+G+β indicate the control group, MSG-treated group, MSG-treated + green-tea-intake group, MSG-treated + β-cryptoxanthin-intake group, and MSG-treated + green tea + β-cryptoxanthin-intake group, respectively. The data are presented as the mean ± standard deviation values. * *p* < 0.05 compared to control animals. # *p* < 0.05 compared to MSG-treated mice.

We analyzed the blood in each group (Figure 2). Blood glucose levels of the MSG group were significantly increased compared to those of the control group (Figure 2A). Blood glucose levels in the groups that consumed green tea catechin or β-cryptoxanthin tended to decrease compared to those in the MSG group; however, the decrease was not significant. Blood glucose levels of the group that took both green tea catechin and β-cryptoxanthin showed a significant decrease compared to those of the MSG group, with values comparable to those of the control group. No significant differences were observed between any group in terms of total protein values (Figure 2B). The total blood lipid level of the MSG group was significantly increased compared to that of the control group (Figure 2C). The total lipid level in the blood of the group ingesting green tea catechin or β-cryptoxanthin tended to decrease compared to that of the MSG group; however, the decrease was not significant. The total lipid level in the blood of the group that took both green tea catechin and β-cryptoxanthin showed a significant decrease compared to that of the MSG group and the control group.

The blood low-density lipoprotein (LDL) cholesterol level of the MSG group did not differ compared to that of the control group; however, the blood LDL cholesterol levels in the other three groups were significantly decreased (Figure 2D). In addition, blood high-density lipoprotein (HDL)-cholesterol levels were compared between the control group and the MSG group, and the blood HDL cholesterol levels of the other three groups showed a decreasing trend (Figure 2E). The free cholesterol level in the blood of the MSG group was significantly increased compared to that of the control group. The free cholesterol level in the blood of the group ingesting green tea catechin or β-cryptoxanthin tended to decrease compared with that of the MSG group; however, the decrease was not significant. The free cholesterol level in the blood of the group that took green tea catechin and β-cryptoxanthin showed a significant decrease compared to that of the MSG group and control group.

The blood free fatty acid levels in the MSG group significantly increased compared to the control group (Figure 2G). Compared to the MSG group, the other three treatment groups showed a significant decrease in blood free fatty acid levels. Blood triglyceride (TG) levels of the MSG group significantly increased compared to those of the control group (Figure 2H). The green-tea-intake group showed a significant increase compared to the control group. However, blood TG levels in the other treatment groups tended to decrease. Blood alkaline phosphatase (ALP) levels of the MSG group were significantly increased compared to those of the control group (Figure 2I). However, the other three treatment groups showed a significant decrease in blood ALP levels compared to the MSG group.

Blood aspartate aminotransferase (AST) levels in the MSG group significantly increased compared to the control group (Figure 2J). In addition, the groups that took green tea catechin alone or β-cryptoxanthin alone showed a significant increase compared to the control group. However, the group that took green tea catechin showed a significant reduction in blood AST levels compared to the MSG group. Furthermore, the group that took both green tea catechin and β-cryptoxanthin showed a significant decrease compared to the MSG group, with values that were comparable to those of the control. Blood alanine aminotransferase (ALT) levels of the MSG group significantly increased compared to those of the control group (Figure 2K). Similarly, the groups that consumed green tea catechin alone or β-cryptoxanthin alone showed a significant increase in blood ALT levels compared to the control and a significant reduction compared to the MSG group. Furthermore, the group that took green tea catechin and β-cryptoxanthin showed a significant decrease compared to the MSG group, with values comparable to those of the control.

### 2.4. Changes in White Adipocytes

**Figure 3:**
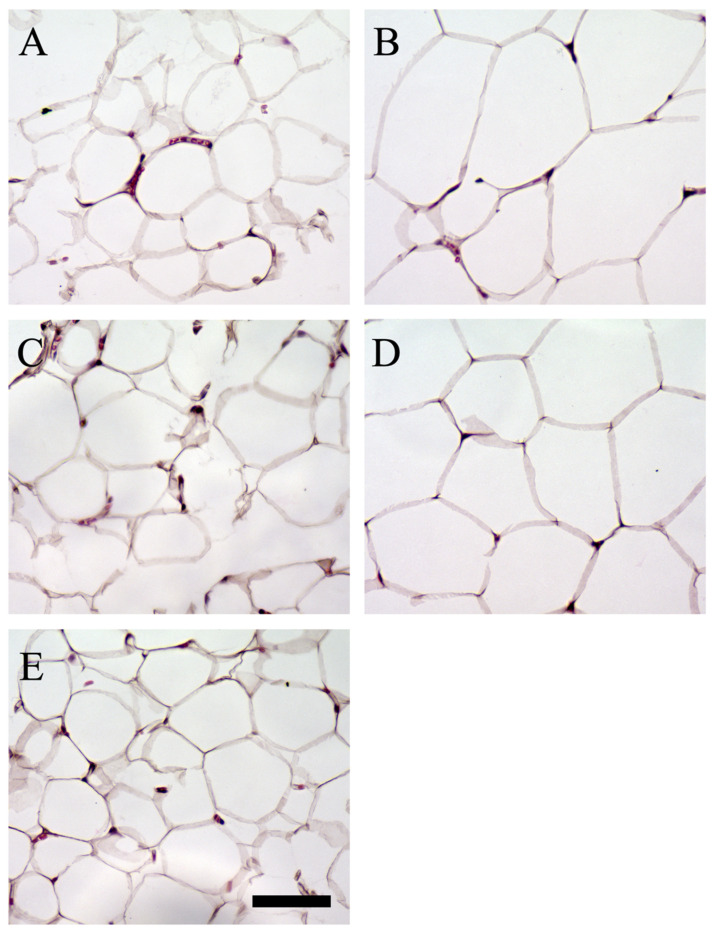
Photographs of white adipocytes. (**A**) Control mice, (**B**) MSG-treated mice, (**C**) MSG + green-tea-intake mice, (**D**) MSG+β-cryptoxanthin-intake mice, and (**E**) MSG + green tea + β-cryptoxanthin-intake mice. Scale bar in (**A**–**E**) = 50 mm.

We investigated whether changes in drinking water caused changes in body fat. White adipocytes present in the abdomen were collected from each animal, weighed, and analyzed histologically. The number of white adipocytes in the mouse body was significantly increased in the MSG-administered group compared to that in the control group (Table 3 and Figure 3). In the groups that consumed green tea catechin or β-cryptoxanthin, no significant decrease was observed compared to the MSG-administered group, and the value remained significantly increased compared to the control group. In contrast, the group that took green tea catechin and β-cryptoxanthin showed a significant decrease in the weight of white adipocytes compared to the MSG-administered group, and this value was similar to that of the control group. The size of white adipocytes in the body was significantly increased in the MSG-administered group compared to that in the control group (Table 3 and Figure 3). In the group that consumed green tea catechin, the size tended to slightly decrease compared to the MSG-administered group; however, the decrease in size was not significant. In addition, in the group that ingested β-cryptoxanthin, the size was maintained at a similar level compared to the MSG group. In the group that ingested the green tea catechin and β-cryptoxanthin, the size of white adipocytes significantly reduced compared to that in the MSG-administered group; the size was similar to that in the control group.

### 2.5. The Adipocyte Inflammatory Response

**Figure 4:**
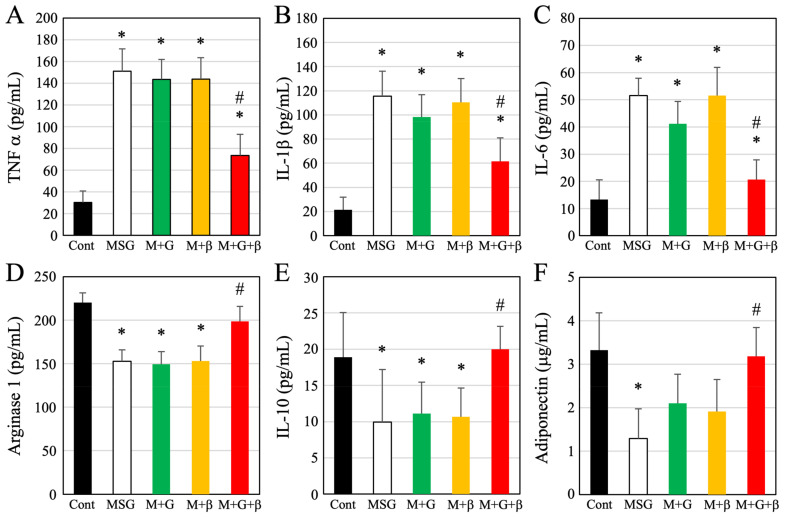
Quantitative analysis of inflammatory cytokines. (**A**) TNF α, (**B**) IL-1β, (**C**) IL-6, (**D**) Arginase 1 (ARG1), (**E**) IL-10, and (**F**) adiponectin. Cont, MSG, M+G, M+β, and M+G+β indicate the control group, MSG-treated group, MSG-treated + green-tea-intake group, MSG-treated + β-cryptoxanthin-intake group, and MSG-treated + green tea + β-cryptoxanthin-intake group, respectively. The data are shown as the mean ± standard deviation values. * *p* < 0.05 compared to control animals. # *p* < 0.05 compared to MSG-treated mice.

Enzyme-linked immunosorbent assays (ELISAs) were used to assess the inflammatory responses within the adipose tissues. Macrophages distributed in adipose tissue and involved in inflammation can be divided into two subtypes: M1 macrophages and M2 macrophages [20,41,42]. First, we examined M1 macrophages that strongly express inflammatory cytokines and are involved in inflammation (Figure 4). TNF-α levels (Figure 4A) were significantly higher in MSG-treated obese mice compared with the normal control. Meanwhile, slight insignificant decreases were observed in the green-tea-intake mice and β-cryptoxanthin-intake mice compared to the MSG-treated mice. In contrast, a significant decrease in TNF α levels was detected in the mice that consumed green tea and β-cryptoxanthin-intake mice. Similarly, interleukin-1β (IL-1β, Figure 4B) and interleukin-6 (IL-6, Figure 4C) levels were also significantly higher in the MSG-treated obese mice compared to the control group. A slight decrease was observed in the levels of IL-1β and IL-6 in the green-tea-intake and β-cryptoxanthin-intake mice, while a significant decrease was detected in the green tea and β-cryptoxanthin-intake mice compared to the MSG-treated mice.

Next, we examined cytokines produced by M2 macrophages. Arginase 1 (ARG1) levels (Figure 4D) were significantly decreased in MSG-treated mice compared to control mice. In the green-tea-intake and β-cryptoxanthin-intake groups, similar levels of ARG1 were detected compared to the MSG-treated group. However, in the green-tea- and β-cryptoxanthin-intake mice, the levels of ARG1 were restored to those of the control group. Similarly, interleukin-10 (IL-10, Figure 4E) levels were significantly lower in the MSG-treated mice compared with the control. Meanwhile, only a slight increase was observed in the green-tea-intake and β-cryptoxanthin-intake groups. In contrast, a significant increase was detected in the green tea and β-cryptoxanthin-intake mice compared with the MSG-treated group, with levels similar to those of the control mice.

Finally, we examined the function of adipocytes. More specifically, the levels of adiponectin—a physiologically active adipokine secreted by adipocytes—were investigated. Adiponectin levels (Figure 4F) were significantly decreased in MSG-treated obese mice compared with the control group. Both the green-tea-intake and β-cryptoxanthin-intake mice showed an upward trend compared with the MSG-treated group; however, the increase was not significant. Meanwhile, the adiponectin levels within the green tea and β-cryptoxanthin-intake mice were significantly increased, with no significant difference compared to the control mice.

## 3. Discussion

Obesity comprises two major metabolic complications associated with poor eating habits and lifestyle. At worst, metabolic problems contribute to many other diseases. There is also an increasing need to control the outbreak of such diseases. Dietary and lifestyle changes contribute to reducing obesity. Recently, a ketogenic diet has been recommended for obesity [43]. However, because of the imbalance in nutrients, it is expected to have a simple obesity control effect. Oxidative stress is enhanced in chronic obesity [44,45,46], which may promote further obesity. Therefore, we postulated that chronic obesity can be prevented or alleviated by reducing oxidative stress. In the present study, the administration of MSG to postnatal mice induced weight gain after weaning (Figure 1). Food intake and water intake indicated that this weight gain was not due to overeating or overdrinking. Other studies suggest that changes in energy metabolism contribute to weight gain. However, compared to the control group, the MSG-administered group showed an increase in daily food intake of approximately 10% (Table 1). The possibility that this non-significant overeating also contributes to weight gain cannot be overlooked.

Feeding is induced by the excitation of neurons in the hypothalamic feeding center associated with a decrease in blood glucose level. After eating, leptin, which is produced as the blood glucose level rises, suppresses the signal for food intake in the hypothalamus and prevents overeating [47,48]. The administration of MSG to mice immediately after birth is believed to cause the following changes. Since the blood–brain barrier is immature immediately after birth, MSG infiltrates the brain and reduces the activity of the arcuate nucleus in the hypothalamus and the nerve cells in the satiety center located near the median eminence [49,50]. Consequently, MSG-treated mice may exhibit hyperphagia and obesity. The elevation of blood glucose caused by food intake, especially carbohydrate intake, increases the influx of carbohydrates into the liver and promotes the synthesis of TGs from carbohydrates. The synthesized TGs are released from the portal vein, increasing the concentration of free fatty acids in the blood; taken up by white adipocytes; and resynthesized and accumulated as TGs in the adipocytes. Additionally, they promote glucose uptake from the blood into adipocytes, promoting lipogenesis. If this situation continues, fat cells become hypertrophied, leading to obesity. In obesity, excess free fatty acids released from adipocytes are taken up by the liver, increasing TG synthesis and causing dyslipidemia. In addition, endogenous cholesterol, such as neutral fat, is primarily synthesized in the liver and transported throughout the body as a complex with lipoproteins and neutral fat in the blood, contributing to various diseases. The changes in blood properties shown in this study likely reflect this effect (Table 1).

Tea catechins are a type of polyphenol abundantly contained in green tea, which has been consumed by people in many countries for many years. Tea catechins, especially gallate-type catechins, taken with meals suppress fat absorption through the inhibition of pancreatic lipase activity in the small intestine and promote fat excretion into feces, thereby reducing body fat. Additionally, gallate-type catechins are associated with bile acid micelles in the small intestine and release cholesterol in the micelles from the micelles. This action suppresses cholesterol absorption in the small intestine and lowers the serum cholesterol level [51]. However, it is believed that tea catechins are absorbed into the body and carried to target cells to act similarly to other physiologically active substances [52]. Tea catechins taken into the body have the effect of enhancing the expression and activity of lipolytic enzymes in adipocytes and increasing the release of adipose-derived glycerol [53]. In addition, they have been found to enhance the activity of β-oxidation-related enzymes in the liver, enhance β-oxidation-related enzymes and fatty acid transport enzymes in skeletal muscles, and increase β-oxidation activity when combined with exercise [54]. Increased fat burning and increased energy consumption in these daily activities are thought to result in an improved energy balance and lipid balance and a reduction in visceral fat. In recent years, it has also been reported that tea catechins enhance the energy metabolism of brown adipocytes, and it is expected that the intake of tea catechins will reduce chronic obesity [55,56].

Among the eight types of catechins, the gallate-type catechins (epigallocatechin gallate, epicatechin gallate, gallocatechin gallate, and catechin gallate) contribute to the effect of tea catechins on moderate fat absorption, while the free-type catechins (epigallocatechin, epicatechin, gallocatechin, and catechin) are not effective. The gallate-type catechin plays a key role in the suppression of fat absorption [57,58]. Long-term intake of gallate-type catechins has been reported to significantly reduce body weight and visceral fat [59]. The tea catechin mix used in this experiment contains a sufficient amount of gallate-type catechins, and its effect on weight control can be considered sufficient (Figure 1). However, the obesity-suppressing effect in humans requires an intake of 200 mg or more per day, equivalent to approximately five cups of ordinary green tea. Currently, although supplements and green tea containing high concentrations of catechins are available on the market, they are not common. Therefore, achieving a synergistic effect with foods other than tea is desirable.

Green tea catechins, which are the bioregulatory components of green tea, exert various physiological actions, such as anticancer, anti-inflammatory, and anti-obesity effects via the 67 kDa laminin receptor (67LR). Reduced expression of 67LR inhibits the effects of green tea catechins [60,61,62]. The expression level of 67LR is regulated by vitamin A and/or E, which is present in several fruits [63,64]. There are plant-derived polyphenols that have been suggested to be related to obesity-reducing effects [65,66]. Therefore, we focused on fruits containing β-cryptoxanthin, which produces an antioxidant effect similar to that of green tea catechins. Xanthophylls, such as β-cryptoxanthin exist in plant bodies as esters bound to fatty acids with 12 to 18 carbon atoms; however, when humans ingest β-cryptoxanthin, the fatty acids are hydrolyzed by intestinal esterase. It is then absorbed as free xanthophyll [67,68]. β-Cryptoxanthin and other carotenoids absorbed from the intestine are incorporated into lipoprotein particles in the liver and transported to various organs through the blood [69,70,71]. β-Cryptoxanthin accumulates again in tissues as a fatty acid ester [67]. Compared with other hydrocarbon-type carotenoids, β-cryptoxanthin has one additional hydroxyl group; therefore, it is believed to be easily absorbed and retained in the body for a long time [34,72]. β-Cryptoxanthin is considered to produce various effects in each tissue, including its antioxidant effect.

Adipose tissue is classified as an endocrine organ that produces and secretes various adipocytokines (physiologically active substances), such as adiponectin, leptin, and TNF-α, in addition to accumulating energy as neutral fat [18,19,73]. Adipose tissue is composed of mature adipocytes and stromal cells; the latter are composed of angiogenic cells, fibroblasts, immune cells, etc., in addition to preadipocytes. Moreover, macrophages are presented within adipose tissue, while obesity causes inflammatory changes in these tissues [21]. The two primary types of macrophages (i.e., M1 and M2) exhibit different properties in adipose tissue [22]. M1 macrophages, which increase with obesity, secrete many pro-inflammatory cytokines and promote inflammatory changes in adipose tissue. Meanwhile, in non-obese adipose tissue, M2 macrophages suppress inflammatory changes by producing anti-inflammatory cytokines. Our ELISA analysis (Figure 4) also confirmed that M1-macrophage-related cytokines significantly increased with obesity (Figure 4A–C). In addition, M2-macrophage-related cytokines were found to be present in control animals, while their abundance was significantly decreased in chronically obese animals (Figure 4D,E). Following the intake of only catechin or only β-cryptoxanthin, neither M1-macrophage-related cytokines or M2-macrophage-related cytokines were significantly affected. In contrast, the intake of both catechin and β-cryptoxanthin significantly decreased the level of cytokines, including TNF-α, associated with M1 macrophages, which increase with obesity. Meanwhile, the abundance of M2-macrophage-related cytokines was also recovered following the intake of both catechin and β-cryptoxanthin. These results indicated that the adipocytes’ inflammatory response was significantly improved with the simultaneous intake of catechin and β-cryptoxanthin. Furthermore, based on the levels of secreted adiponectin, we postulated that adipocytes exhibited improved pathological changes in adipose tissues caused by chronic obesity following combined intake. Improving adiponectin levels is thought to enhance fatty acid combustion and glucose uptake, enhance insulin sensitivity, and improve metabolic syndrome [74,75].

The bioregulatory function of food is expected to aid in preventing and improving disease states, including rapidly increasing lifestyle-related diseases. This strategy is evidenced by the active development of functional foods around the world. Similar to our report, another report has examined the effects of the food pairing of green tea and citrus-derived polyphenols in humans [76]. In this cohort study, long-term (12 weeks) intake has been reported to reduce body weight, improve BMI, and improve blood biochemical tests. This study suggested that polyphenols derived from citrus fruits, such as mandarin oranges, improve the function of green tea catechins. In our study, we used mandarin oranges containing β-cryptoxanthin, which is expected to exert a stronger antioxidant effect than citrus-derived polyphenols. The ingestion of green tea and mandarin oranges containing β-cryptoxanthin together in a short period (4 weeks) is expected to elicit an anti-obesity effect with a smaller amount of green tea catechin intake than that previously reported. Since this amount can easily be consumed in a single day, this strategy is expected to be useful for reducing obesity.

In humans, obesity can be induced by overeating, as well as by changes in diet, especially the intake of high-calorie foods. Since the obesity model mouse used in this study is not an obesity model of high calorie intake, it is difficult to clarify whether it would have the same effect on human obesity. Further investigation is necessary to examine whether the findings obtained in this study have a preventive effect on obesity associated with high-calorie intake. However, the results of this study are expected to support further investigation of the mechanism underlying the anti-obesity effect of tea catechins, detailed elucidation of the food pairing effect of green tea catechins and citrus-derived ingredients, and empirical research in humans.

## 4. Materials and Methods

### 4.1. Animals

Male C57BL/6J mice were used in this study. C57BL/6J mice (five mother mice with eight male pups) were purchased from Japan SLC Co., Ltd. (Shizuoka, Japan). Until weaning (3 weeks old), they were housed in one cage with their mothers. After 3 weeks of age, two mice were housed per cage until the day of the experiment under a constant room temperature (23 ± 2 °C), humidity (55 ± 10%), and 12 h light–dark cycle while having ad libitum access to drinking water and food.

This study was approved by the Laboratory Animal Ethics Committee of Meiji Pharmaceutical University (No. 2707, 1 April 2020–2022). In accordance with the Meiji Pharmaceutical University Experimental Animal Guidelines, we reduced the number of animals as much as possible and minimized their suffering.

### 4.2. Producing Obese Mice

Based on previous research reports [14,15,16], we produced a mouse model with chronic obesity. MSG (Wako Pure Chemical Industries Ltd., Tokyo, Japan) was injected subcutaneously at 2 mg/body weight into 32 male mice at 1, 2, 4, 6, 8, and 10 days of age (MSG-administered group). Eight animals, which comprised the control group, were subcutaneously injected with the same volume of saline. Weight gain after weaning was measured.

### 4.3. Weight Measurement, Measurement of Food and Water Intake, Drinking of Green Tea and/or β-Cryptoxanthin

After 3 weeks of weaning, normal mice and MSG-administered mice were weighed every week at a fixed time (noon). The amount of food ingested in each cage was measured every day (week 11 to week 15) at a fixed time (noon), and the average daily amount of food consumed per animal was calculated. Similarly, the amount of water ingested in each cage was measured every day (week 11 to week 15) at a fixed time (noon), and the average daily amount of water consumed per animal was calculated.

Green tea components and/or β-cryptoxanthin was administered by free drinking [38,77] for 4 weeks from week 11 to week 15 (Table 4). The green tea catechin mix (Sigma-Aldrich Japan, Tokyo, Japan) was dissolved in water and administered (1.7 mg/kg) daily at half the concentration that showed efficacy in humans (200 mg/60 kg ideal human body weight) [30,31]. Green tea catechin contains caffeine, catechin, catechin 3-gallate, epicatechin, epicatechin-3-gallate, epigallocatechin 3-gallate, gallocatechin, and gallocatechin 3-gallate according to the manufacturer’s package insert. Similarly, β-cryptoxanthin (Wako Pure Chemical Industries Ltd., Tokyo, Japan) was dissolved in water, and 50 μg/kg body weight was ingested daily, as in our previous study [38].

### 4.4. Blood Biochemistry Test

Blood was collected from all 15-week-old mice in each group and gently mixed by inversion. Subsequently, the cells were allowed to stand at room temperature for 30 min to confirm blood coagulation and centrifuged at 1500× *g* for 20 min to obtain the serum, which was placed in a separate tube and rapidly frozen. The appropriate test kits (Wako Pure Chemical Industries Ltd., Osaka, Japan) were used to determine the levels of glucose, total protein, total lipid, LDL cholesterol, HDL cholesterol, free cholesterol, free fatty acid (non-esterified fatty acid), TG (neutral fat), ALP, AST, and ALT.

### 4.5. Histological Analysis

Following blood collection, the mice were deeply anesthetized, and internal adipose tissues were collected. After their weight was measured, half of the adipose tissues were immersed and fixed in a 4% paraformaldehyde solution (pH 7.4) for 2 days. The immersion-fixed adipose tissue was washed with phosphate buffer, immersed in a dehydration series (50, 70, 80, 90, 95, and 100% ethanol), which was subsequently replaced with Lemosol A (Wako Pure Chemical Industries Ltd., Tokyo, Japan), and embedded in paraffin. The paraffin-embedded adipose tissue was sliced with a sliding microtome (REM-710; Yamato Kohki Industrial, Tokyo, Japan) into 5 μm thick slices, deparaffinized, and stained with hematoxylin–eosin staining solution (Muto Pure Chemicals Co. Ltd., Tokyo, Japan). After washing, the sections were dehydrated using graded concentrations of ethanol and Lemosol A (Wako Pure Chemical Industries Ltd., Tokyo, Japan), and a cover slip was placed. The tissue sections were examined using an optical microscope (BZ-X700; Keyence, Osaka, Japan), and images were obtained.

The area of white adipocytes was measured using the Image J software. Analysis was performed using five photographs from one mouse, with eight animals in each group.

### 4.6. ELISA Analysis

Each mouse in each group was used. Their adipose tissues were rapidly homogenized in ice-cold Cell Extraction Buffer (Abcam, Cambridge, UK). Homogenates were incubated on ice for 20 min, centrifuged at 18,000× *g* for 20 min at 4 °C, and thoroughly rinsed in PBS to remove blood. Supernatants were transferred to clean tubes, and the pellets were discarded. Samples were either used immediately in assays or were aliquoted and stored at −80 °C.

Samples were processed using the mouse Arginase 1 ELISA Kit (Abcam, Cambridge, UK), mouse TNF alpha ELISA Kit (Abcam, Cambridge, UK), mouse IL-6 ELISA Kit (Abcam, Cambridge, UK), mouse IL-10 ELISA Kit (Abcam, Cambridge, UK), mouse IL-1 beta ELISA Kit (Abcam, Cambridge, UK), and mouse adiponectin ELISA Kit (Abcam, Cambridge, UK). Briefly, a total of 50 μL of all samples was added to appropriate wells, and 50 μL of each antibody was added and mixed. Plates were sealed and incubated for 1 h at room temperature on a plate shaker set to 400 rpm. Each well was then washed with 3 × 350 μL of 1× Wash Buffer PT. Next, 100 μL of TMB Development Solution was added to each well and incubated for 10 min in the dark on a plate shaker set to 400 rpm. Stop Solution (100 μL) was then added to each well and mixed on a plate shaker for 1 min. Using a microplate reader (Infinite F50 plus, Tecan, Zürich, Switzerland), the optical density was measured at 595 nm.

### 4.7. Statistical Analysis

The data obtained from each experiment are shown as the mean ± standard deviation values. Differences were analyzed using analysis of variance. Significance was set at *p* < 0.05. Statistical analysis was performed using Excel software (Microsoft Corp., Redmond, WA, USA) and StatView statistical software (version 5.0.1, SAS Institute Inc., Cary, NC, USA).

## 5. Conclusions

Collectively, these results suggest that the combination of tea catechins and antioxidant foods can alleviate chronic obesity, indicating that a combination of various ingredients in foods might contribute to reducing chronic obesity.

## Figures and Tables

**Table 1:** Changes in the amount of food intake (g/day) from 11 weeks to 15 weeks of age.

Experimental Groups	11 w–12 w	12 w–13 w	13 w–14 w	14 w–15 w
Control	4.29 ± 1.27	4.22 ± 1.07	4.31 ± 1.77	4.40 ± 1.28
MSG	4.68 ± 1.61	4.51 ± 1.33	4.51 ± 1.40	4.57 ± 1.67
MSG + green tea catechin	4.59 ± 1.62	4.61 ± 1.40	4.59 ± 1.25	4.55 ± 1.66
MSG + β-cryptoxanthin	4.71 ± 1.45	4.60 ± 1.23	4.39 ± 1.38	4.62 ± 1.53
MSG + green tea + β-cryptoxanthin	4.57 ± 1.38	4.52 ± 1.02	4.45 ± 1.71	4.48 ± 1.32

The data are shown as the mean ± standard deviation values.

**Table 2:** Changes in drinking volume (mL/day) from 11 weeks to 15 weeks of age.

Experimental Groups	11 w–12 w	12 w–13 w	13 w–14 w	14 w–15 w
Control	4.13 ± 0.10	4.20 ± 0.26	4.23 ± 0.19	4.25 ± 0.17
MSG	4.48 ± 0.15	4.43 ± 0.17	4.48 ± 0.25	4.38 ± 0.15
MSG + green tea catechin	4.18 ± 0.05	4.40 ± 0.08	4.38 ± 0.15	4.40 ± 0.08
MSG + β-cryptoxanthin	3.95 ± 0.13	4.38 ± 0.21	4.55 ± 0.13	4.53 ± 0.22
MSG + green tea + β-cryptoxanthin	4.20 ± 0.08	4.40 ± 0.14	4.50 ± 0.08	4.53 ± 0.05

The data are expressed as the mean ± standard deviation values.

**Table 3:** Changes in visceral white adipose tissue at 15 weeks of age.

Experimental Groups	Weight (g)	Average Diameter (mm^2^)
Control	0.8 ± 0.2	1457.2 ± 763.0
MSG	2.4 ± 0.5 *	5922.7 ± 3211.9 *
MSG + green tea catechin	1.9 ± 0.6 *	4197.8 ± 2741.3 *
MSG + β-cryptoxanthin	2.3 ± 0.5 *	5558.9 ± 873.8 *
MSG + green tea + β-cryptoxanthin	0.9 ± 0.7 #	1622.5 ± 1081.2 #

The data are shown as the mean ± standard deviation values. * *p* < 0.05 versus control value. # *p* < 0.05 versus MSG value.

**Table 4:** Experimental animals (8/group, experimental age = 15 weeks).

Experimental Groups	Drinking Period
Control	-
MSG	-
MSG + green tea catechin	11–15 w
MSG + β-cryptoxanthin	11–15 w
MSG + green tea + β-cryptoxanthin	11–15 w

## Data Availability

The datasets used and/or analyzed during this study are available from the corresponding author upon reasonable request.
